# Conversion therapy for advanced hepatocellular carcinoma with vascular invasion: a comprehensive review

**DOI:** 10.3389/fimmu.2023.1073531

**Published:** 2023-04-26

**Authors:** Zunyi Zhang, Erlei Zhang

**Affiliations:** Research Laboratory and Hepatic Surgery Center, Department of Hepatic Surgery, Tongji Hospital, Tongji Medical College, Huazhong University of Science and Technology, Wuhan, China

**Keywords:** conversion therapy, HCC, advanced stage, vascular invasion, downstage

## Abstract

Hepatocellular carcinoma (HCC) is the most common type of liver cancer and has a high mortality rate worldwide. The percentage of HCC patients with vascular invasion at the time of initial HCC diagnosis is 10%–40%. According to most guidelines, HCC with vascular invasion is classified as advanced stage, and resection is only suggested for a minority of such patients. Recently, advances in systemic and locoregional treatments for such patients have resulted in amazing response rates. Therefore, a “conversion therapy” strategy including systemic and locoregional treatments is proposed to select patients from an initially unresectable state to eventually undergo R0 resection. Recently, many studies have proven that conversion therapy followed by subsequent surgery is achievable in well-selected advanced HCC patients and can provide prolonged long-term outcomes. Based on published research, this review has summarized the clinical experience and evidence of conversion treatment in HCC patients with vascular invasion.

## Introduction

Hepatocellular carcinoma (HCC) is the most common type of liver cancer and has a high mortality rate worldwide. The percentage of HCC patients with vascular invasion at the time of initial HCC diagnosis is 10%–40% (1–3). According to the American Association for the Study of Liver Disease/Barcelona Clinic for Liver Cancer (AASLD/BCLC) staging system and treatment guidelines, HCC associated with vascular invasion or bile duct invasion is regarded as an advanced stage. The suggested treatment for such patients is systematic treatment or conservative treatment (4). However, the median survival time of these patients is very unsatisfactory. According to the China Liver Cancer Staging (CNLC) system (5), HCC with vascular invasion is regarded as advanced IIIa stage. In contrast to Western countries, hepatectomy and locoregional therapies, including transarterial chemoembolization (TACE), HAIC, or radiation therapy combined with systemic treatment, are suggested for HCC patients with macrovascular invasion. Compared with systematic treatment or conservative treatment only, combined therapy might provide a better prognosis with minimal side effects (6).

Downstaging conversion therapy is a new strategy for unresectable HCC that aims to reduce tumor burden by a combination of locoregional or systemic therapy and eventually allow patients to become amenable to surgical resection. The effects of this type of surgical resection are still under debate. Many concerns about downstaging conversion therapy remain. This review article discusses the following problems in downstaging conversion therapy. First, the target patients are chosen; second, the most commonly used conversion downstaging treatment and its conversion rate are introduced; and third, a possible hypothesis to increase the efficacy of conversion treatment is proposed.

## The target population of conversion downstaging treatment for HCC patients with vascular invasion

As we have mentioned above, conversion downstaging treatment is proposed for unresectable HCC patients. The major reasons for unresectable HCC with vascular invasion can be divided into surgical and oncological causes (7). Surgical causes are common for most surgeons, which means that surgical excision cannot be performed safely because of the patient’s general condition, liver function, or insufficient remnant liver volume. Oncological causes refer to the prognosis after hepatectomy failing to surpass other nonsurgical treatments (7, 8). This part of the cause is still under debate between surgeons and medical oncologists. Vascular invasion in HCC patients includes two different types, i.e., tumor thrombosis in the portal vein (PVTT) or in the hepatic vein (HVTT) (9, 10). Depending on the different locations of the tumor thrombus, the complications and prognosis of surgery or nonsurgical treatment might be different. Based on recent reports focused on comparing the prognosis between hepatectomy and nonsurgical treatment, we tried to find the target population of HCC with vascular invasion that is not suitable for hepatectomy because of oncological reasons.

**Figure 1:**
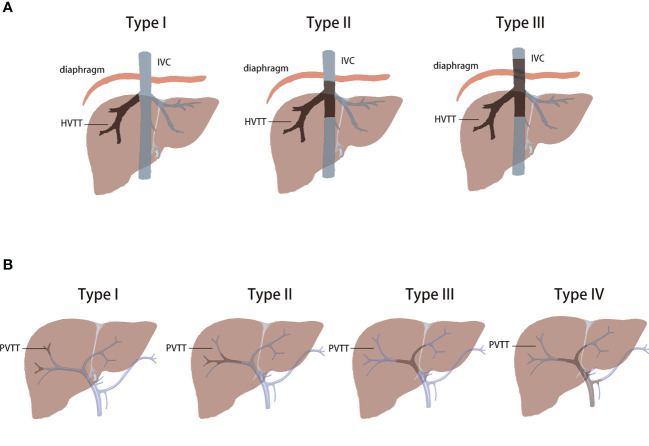
**(A)**, classification of the tumor thrombus in the portal vein; **(B)**, classification of the tumor thrombus in the hepatic vein (14, 15).

To date, the survival rate is poor for HCC patients with macrovascular invasion. Surgical treatment is generally not suggested because tumor cells might spread throughout the whole body, and the survival time is only 2.7–4 months after diagnosis if no suitable treatment is adopted (11). However, the prognosis of HCC with vascular invasion varies according to the different locations of vascular invasion. As we have mentioned above, vascular invasion in HCC patients can be divided into two different types: PVTT and HVTT. Compared with HCC patients with PVTT, HCC patients with only HVTT have different outcomes (12). The prognosis of HCC patients with either type of vascular invasion is correlated with the extent of invasion (9, 13, 14). Based on the extent of invasion, PVTT could be divided into type I–IV and HVTT could be divided into type I–III according to Chen’s study (14, 15) (**Figure 1**). For HCC patients with vascular invasion in the peripheral hepatic vein, the overall survival (OS) after hepatectomy might be 27.1–63 months (12–14). If vascular invasion in the hepatic vein extends into the inferior vena cava/right atrium, the OS after hepatectomy might be shortened to 5–16.8 months (13, 14, 16, 17). For HCC patients with vascular invasion in the intrahepatic portal vein, after R0 resection, the OS might be 18–50 months (2, 13, 18). If vascular invasion in the portal vein extends into the main portal vein, the OS after hepatectomy might be only 6–10 months (3, 13, 15). In some published research, resection might provide a better prognosis than nonsurgery treatment, including TACE/HAIC or sorafenib treatment, in HCC patients with PVTT (13). Nevertheless, when subgroup analysis was performed, the prognosis after hepatectomy combined with thrombectomy seemed to not surpass the prognosis after nonsurgical treatment if the tumor thrombus had already extended into the main portal vein (13, 19). Therefore, for HCC patients with PVTT, if the intrahepatic tumor lesion is resectable, main portal vein thrombus might be an indication for conversion therapy. For HCC patients with HVTT, the situation might be different. According to Kokudo’s study, the mean survival time in HCC patients with HVTT in the major hepatic vein was similar to that in patients with HVTT in the peripheral hepatic vein after hepatectomy (4.85 years vs. 4.67 years, respectively). However, in the nonsurgery group, the mean survival time was only 1.58–1.81 years. As PVTT is usually accompanied in HCC patients with HVTT, it could greatly decrease the mean survival time (9, 12). Therefore, for HCC patients with HVTT, conversion therapy might be suggested if the HVTT has already extended into the inferior vena cava or has been accompanied by PVTT.

Since treatment options for HCC have been studied, such as tyrosine kinase inhibitor (TKI) treatment combined with immune treatment, many clinical trials have been carried out in advanced HCC patients. Many amazing results have been published based on TKI treatment and immune treatment combined with or without locoregional treatment. For example, in a clinical trial (Keynote 524) (20), lenvatinib (a kind of TKI medicine) combined with pembrolizumab provided an OS of 22 months in unresectable HCC patients, among which 79% were BCLC-C stage HCC patients. When combined with locoregional treatment, including TACE/HAIC/radiation therapy, the 1-year survival rate of unresectable HCC patients is extended to 75%–100% (21–24). However, thus far, there are still no well-designed clinical trials for HCC patients with vascular invasion to compare the prognosis after hepatectomy or nonsurgical treatment.

Based on published data and the China liver cancer staging system (5), the prognosis of HCC patients with vascular invasion after hepatectomy varies according to the extent of vascular invasion. Resection cannot surpass nonsurgical treatment if vascular invasion is detected in the main portal vein (PVTT type III and IV) and inferior vena cava (HVTT type II and III). Therefore, for these patients, conversion therapy should be suggested even if the tumor and thrombus can be resected. With the improvement of systemic therapy, the target population of conversion therapy in HCC patients with vascular invasion might be further enlarged.

## Conversion therapy in advanced-stage HCC with vascular invasion

The principle of conversion therapy for advanced-stage HCC with vascular invasion is to downstage HCC patients by utilizing multiple treatments. The most commonly used treatments for conversion therapy are systemic treatments combined with or without locoregional treatments. Locoregional treatments include transcatheter arterial chemoembolization (TACE), hepatic arterial infusion chemotherapy (HAIC), and radiation therapy. Systemic treatments should include TKIs, immunotherapy, and chemotherapy. To downstage HCC patients in a short time, a combination of multimodality treatment approaches is usually suggested. In the following article, we summarize the reported data concentrated on the conversion treatment of HCC patients with vascular invasion according to different multimodality combinations.

Sorafenib is the first TKI inhibitor that has been demonstrated to have a survival benefit in advanced HCC (25). However, the prognosis of HCC after sorafenib has been associated with a low objective response rate (ORR) of 3.3% (25). At the same time, locoregional treatments for HCC patients with vascular invasion, such as TACE or HAIC, have shown limited efficacy (**Table 1**). Although some of the reports showed that conversion could be performed in such patients, the conversion rate was lower than 15%, which is not satisfactory (34). Therefore, for a long time, neoadjuvant and downstaging therapy could not be suggested as a standard protocol for advanced HCC. In 2018, the phase 3 REFLECT trial showed that lenvatinib was associated with an amazing ORR of 40.6% in unresectable HCC patients (36). Although lenvatinib did not provide better OS than sorafenib, it brings us a new hope of conversion therapy. In the following years, some new TKI treatments, such as donafenib and regorafenib, have proven their value in advanced HCC, although no clinical studies have been reported. In a parallel-controlled phase II–III trial, donafenib showed a better ORR than sorafenib (4.6% versus 2.7%, respectively) (37). In the donafenib group, 0.3% of patients had a complete response, and 5.8% of patients had a partial response, whereas no patients had a complete response and only 3% of patients had a partial response. Regorafenib is an oral TKI shown to provide a survival benefit in HCC patients progressing on sorafenib. The ORR was 11% in patients progressing on sorafenib. One percent of patients had a complete response (38). With amazing efficacy compared with sorafenib, new TKI drugs provide new insight into conversion treatment.

**Table 1:** Recent clinical trials concentrated on conversion therapy.

Study	TKI treatment	Immune therapy treatment	Locoregional therapy	ORR	Conversion rate*	Time for maintenance therapy	OS
Zhu et al. (26)	Lenvatinib/apatinib	Pembrolizumab/camrelizumab	None	54.2%	7/34	4–6 weeks	Over 11 months
Wu et al. (27)	Lenvatinib	Various	TACE	77.4%	19/35	3–6 months	Not reached
Shindoh et al. (28)	Lenvatinib	None	None	63.6%	4/12	Not mentioned	27.4 months
Zhang et al. (29)	Lenvatinib/apatinib	Sintilimab/camrelizumab	HAIC	96%	60%	Not mentioned	Over 12.53 months
Kaneko et al. (30)	Sorafenib/lenvatinib	None	Radiotherapy/TACE	24%	3/130	Not mentioned	Not reached
MK et al. (31)	Sorafenib	None	HAIC	40%	5/35	Not mentioned	Not mentioned
Huang et al. (6)	Sorafenib	Camrelizumab	TACE and SBRT	41.7%	4/13	Not mentioned	Over 1 year
Rana et al. (32)	Sorafenib	None	Yttrium-90 radioembolization	30%	1/10	At least 8 months	Not reached
Zhang et al. (33)	None	None	TACE	Not mentioned	12 conversed	Not mentioned	58 months
Lee et al. (34)	None	None	HAIC	36.3%	10/67	None	37 months
Chong et al. (35)	None	None	HAIC and radiation	69.2%	26/98	None	Not mentioned

* Conversion rate is expressed as converted HCC patients over total patients with vascular invasion.

Immune checkpoint inhibitors (ICIs) are now being introduced into HCC treatment. Although it has not been placed in the first-line treatment, positive outcomes have been achieved in the combination of TKI treatment and ICIs in conversion treatment when compared with sorafenib treatment (26). In one phase Ib single-arm study of 100 unresectable HCC patients, lenvatinib plus pembrolizumab yielded a confirmed response rate of 46% according to mRECIST (20). Among these patients, 20% had macroscopic vascular invasion. Although no further report on the conversion rate in patients with vascular invasion was published, the high ORR rate still provides insight into conversion for HCC patients with vascular invasion (7). Recently, a phase III study of lenvatinib plus pembrolizumab versus lenvatinib for advanced HCC patients was reported (39). The OS and progression-free survival (PFS) were not significantly different between these two groups. However, in the subgroup analysis of OS, the HR in the macrovascular invasion/extrahepatic spread or HBV etiology group indicated that advanced HCC patients could benefit from lenvatinib plus pembrolizumab (39). According to Zhu’s study, lenvatinib plus a PD-1 inhibitor showed an ORR of 54.3%, and 20% of HCC patients with vascular invasion could undergo surgery (30). Except for the combination of TKI treatment and PD-1 inhibitors, VEGFR inhibitors (bevacizumab) combined with PD-L1 inhibitors (atezolizumab) showed a better prognosis than sorafenib in unresectable patients (32). Although no reported data on bevacizumab combined with atezolizumab have been reported in HCC patients with vascular invasion, the promising ORR of 26% in unresectable HCC shows good insight into its usage in conversion therapy.

Various combinations of treatments have been used in unresectable HCC. Locoregional therapy included HAIC, TACE (40), transarterial radiotherapy (TARE) (29), and radiotherapy. TACE and HAIC are two different treatments that take advantage of the relatively selective arterial vascularization of hepatic tumors (41, 42). More than one guideline has suggested that TACE/HAIC could be applied in patients with vascular invasion (5, 43). However, because of different characteristics between HVTT and PVTT, the prognosis for these patients after TACE and HAIC might be different. To date, most reports based on HAIC and TACE have mainly focused on PVTT (33, 44–46). In HCC patients with PVTT, HAIC could provide a high response rate between 36% and 50% (45, 47). At the same time, TACE could also provide a response rate between 13% and 33% (33, 35, 48). The ORR of TACE and HAIC applied to HCC patients with PVTT is acceptable, so several studies have been proposed to check whether TACE or HAIC could be applied in conversion therapy alone (**Table 1**). However, the conversion rate reported was 14% to 26%, which was significantly lower than that of combined treatment (34, 49).

As TKI inhibitors or VEGFR inhibitors could inhibit the vascular endothelial growth factor receptor, whose level could be elevated after TACE or HAIC, systemic treatment is believed to enhance the efficacy of TACE or HAIC in HCC patients with PVTT (27, 31). When combined with systemic treatment, the ORR of HAIC or TACE could be elevated to 40%–77.4% (28, 48, 50). The successful conversion rate for unresectable HCC patients with vascular invasion could be increased to 14%–33% (50, 51) (**Table 1**). ICIs are a new kind of therapy that could enhance the efficacy of TKI inhibitors. The combination of TKI inhibitors, ICIs, and TACE or HAIC has also been introduced into unresectable patients with vascular invasion. The ORR of patients has been increased to 77%–96%, which was amazing (28, 42). The conversion rate from an unresectable state to a resectable state could be increased to 54%–60% (28, 42). As we have discussed in the former part, the study reported conversion therapy in HCC with vascular invasion primarily concentrated in portal vein invasion. To date, no specific study has focused on the efficacy of conversion therapy in unresectable HCC patients with HVTT. Because of the unique characteristics of HVTT, the efficacy of conversion therapy in HCC patients with HVTT might be different, which needs further discussion. According to **Table 1**, for successfully converted HCC patients, the existing studies usually recommend prolonged systemic therapy for more than 6 months. However, this suggestion was proposed only based on the experience of the surgeon. The time for maintenance therapy still needs further confirmation by well-designed clinical trials.

## Hypothesis on how to increase the efficacy of conversion treatment

As we have mentioned above, HCC is usually diagnosed in advanced stages, which has limited treatment options. Before systemic therapy was introduced, conversion therapy was restricted to TACE or HAIC combined with or without sorafenib. Because of the low ORR of these treatments, the conversion rate for HCC with advanced stage is not satisfactory (**Table 1**). Therefore, seeking new combination treatment strategies to increase the conversion rate in advanced-stage HCC is a major challenge. Current clinical trials emphasize the breakthrough of lenvatinib combined with pembrolizumab in unresectable HCC (20). Despite the amazing ORR, some patients still have no response. To further increase the ORR of HCC patients in advanced stages, the mechanism needs to be clarified for optimal patient selection or the other adjuvant therapies need to be combined with TKI treatments and ICIs to enhance the treatment efficacy.

Various immunotherapeutic clinical trials have been conducted for HCC, but there is still limited evidence of HCC biomarkers to select HCC patients. In general, PD-1/PD-L1 expression, microsatellite instability, tumor mutation burden, and immunosuppressive cells, including tumor-associated macrophages, marrow-derived suppressor cells, and regulatory T cells (Tregs), are used as biomarkers associated with immunotherapy effects in various cancer types (52, 53). In Yi’s report (54), lenvatinib reduced tumor lesion PD-L1 levels and Treg differentiation to improve anti-PD-1 efficacy by blocking FGFR4. Levels of FGFR4 expression and Treg infiltration in HCC tissues could function as biomarkers to screen sensitive patients with lenvatinib plus anti-PD-1 combination therapy. Chronic liver injury exists in most HCC patients and can lead to liver inflammation, fibrosis, and eventually cirrhosis. Liver cirrhosis is also a state of immune dysfunction caused by excessive activation of proinflammatory cytokines (55). In patients with cirrhosis, an abnormal gastrointestinal barrier and bacterial translocation could increase the risk of infection and sepsis in the abdomen and carcinogenesis (56). Shi’s study showed that commensal probiotics combined with IL-2-based immunotherapy could enhance the antitumor immune response and tumor clearance (56). Microsatellite instability and tumor mutation burden in HCC are rare in HCC and have not yet been applied in the clinic. In recent years, genomics and bioinformatics have enabled the identification of target populations of HCC patients. Several signatures have been proposed to predict the prognosis of HCC patients based on lncRNAs, miRNAs, and mRNAs (57–59). According to Dai’s report, an immune-related gene-based prognostic index containing 11 differentially expressed immune-related genes was constructed by using a LASSO regression model to predict the infiltration of immune cells in the tumor microenvironment of HCC and predict the response to immune therapy at the same time (59). However, the application of these results in the clinic is still limited.

**Figure 2:**
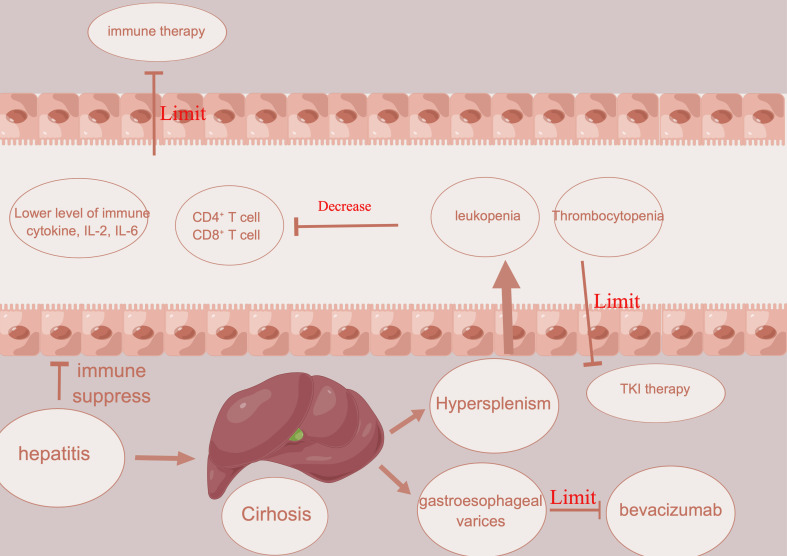
Illustration about how hepatitis, cirrhosis, and portal hypertension limit the usage of immune therapy and angiogenesis inhibitors.

Portal hypertension is usually detected in HCC patients in Asia due to chronic viral hepatitis and cirrhosis (60). It often presents with gastroesophageal varices and hepatic encephalopathy, hypersplenism, thrombocytopenia, and leukopenia (**Figure 2**). With the complication mentioned above, many treatments could be limited in such patients. In HCC patients with vascular tumor thrombus, there is usually a higher chance of worse hepatic function and complications related to portal hypertension, which eventually affect the usage of treatment (61). For example, atezolizumab plus bevacizumab showed a better prognosis than sorafenib in unresectable patients (32). This highlights the usage of this kind of combination in conversion therapy. However, bevacizumab, a kind of VEGFR inhibitor, has a high risk of gastrointestinal bleeding (62). Therefore, key exclusion criteria for this treatment included untreated or incompletely treated esophageal or gastric varices with bleeding or a high risk of bleeding (62). In addition to bleeding, which could restrict the use of possible conversion therapy in HCC patients with vascular invasion, thrombocytopenia is another restriction for conversion therapy in HCC patients with vascular invasion. Thrombocytopenia is a common complication in HCC patients with portal hypertension and is caused by decreased levels of thrombopoietin and increased levels of platelet destruction by hypersplenism (63, 64). In HCC patients with vascular invasion, the incidence of thrombocytopenia might be increased further (65). For the most commonly used TKI inhibitors, including sorafenib or lenvatinib, a platelet count decrease is detected in nearly 20% of HCC patients (66, 67). Once a platelet count decrease is detected in HCC patients with thrombocytopenia, a dose reduction or withdrawal would be needed, which would then decrease the efficacy of conversion therapy. Several reports have reported that hypersplenism could downregulate the immune function of the spleen in the development of HCC (68). Hypersplenism could lead to an abnormal T-cell receptor CD3 complex and suppress the immune function of T cells (69). Except for the T-cell receptor CD3 complex, splenic CD11b+Gr-^int^Ly6^chi^ cells expand in the marginal zone of the spleen, which is associated with memory CD8+ T cells, cross-presenting tumor antigens and eventually leading to immune suppression (70). Although there is still no report of the prognosis between hypersplenism and the efficacy of immune therapy, leukopenia in HCC patients with vascular invasion and hypersplenism might restrict the efficacy of immunotherapy or conversion therapy.

Splenectomy has been performed in cirrhotic patients since 1950 and can address portal hypertension, thrombocytopenia, and leukopenia (71). Several studies have suggested that splenectomy could improve the prognosis of HCC in patients (70, 72). First, splenectomy could reduce portal vein pressure and increase the level of white blood cells and platelet counts, which could lower the risk of restriction of conversion therapy (73). Second, splenectomy could promote recovery of the balance between T lymphocyte subsets and improve antitumor immunology (74). After splenectomy, natural killer cells and CD4+ and CD8+ cells are increased, which could increase the immune response against HCC (75). To increase the efficacy of conversion therapy, splenectomy might be a good choice. Except for splenectomy, partial splenic embolization (PSE) can function similarly to splenectomy. After PSE, white blood cells (neutrophils, lymphocytes, and monocytes) could also increase. Th1 and Th2 cells could also be increased compared with those before PSE, which means that PSE could not only promote the recovery of leukopenia and thrombocytopenia but also induce activation of host immunity (76).

## Conclusion

Compared with other treatments, surgery is still the only curative treatment (13). However, only a minority of patients with HCC receive curative treatment. Conversion therapy aimed at tumor downregulation could improve the prognosis in HCC patients with vascular invasion (77). In the era of immune therapy combined with targeted therapy, the ORR is elevated in HCC patients with vascular invasion. Despite this, an increasing number of reports have suggested that locoregional treatment, including TACE or radiotherapy combined with systemic therapy, could increase the conversion rate for advanced HCC patients. Because of the specialty of HCC patients with vascular invasion, patients with PVTT or HVTT should be considered under different situations. Since reports on conversion therapy in HCC patients with vascular invasion are limited, the conversion rate is still unknown. Hypersplenism, as a common state in HCC patients with vascular invasion, is a type of restriction in conversion therapy. To improve the conversion rate in HCC patients with vascular invasion, clarifying the mechanism for optimal patient selection and adjuvant splenectomy might be helpful. Currently, the outcomes for patients who have chosen conversion therapy are promising, and the data from well-designed large randomized controlled trials are still limited and require further investigation.

## Author contributions

ZZ performed the majority of the writing and prepared the table. EZ designed the outline of this paper. All authors contributed to the article and approved the submitted version.
